# Structural features in the glycine-binding sites of the GluN1 and GluN3A subunits regulate the surface delivery of NMDA receptors

**DOI:** 10.1038/s41598-019-48845-3

**Published:** 2019-08-23

**Authors:** Kristyna Skrenkova, Katarina Hemelikova, Marharyta Kolcheva, Stepan Kortus, Martina Kaniakova, Barbora Krausova, Martin Horak

**Affiliations:** 1 Institute of Experimental Medicine of the Czech Academy of Sciences, Videnska 1083, 14220 Prague 4, Czech Republic; 2 Institute of Physiology of the Czech Academy of Sciences, Videnska 1083, 14220 Prague 4, Czech Republic; 3 Department of Physiology, Faculty of Science, Charles University in Prague, Albertov 6, 12843 Prague 2, Czech Republic

**Keywords:** Ion channels in the nervous system, Molecular neuroscience

## Abstract

*N*-methyl-D-aspartate receptors (NMDARs) are ionotropic glutamate receptors that play an essential role in mediating excitatory neurotransmission in the mammalian central nervous system (CNS). Functional NMDARs are tetramers composed of GluN1, GluN2A-D, and/or GluN3A-B subunits, giving rise to a wide variety of NMDAR subtypes with unique functional properties. Here, we examined the surface delivery and functional properties of NMDARs containing mutations in the glycine-binding sites in GluN1 and GluN3A subunits expressed in mammalian cell lines and primary rat hippocampal neurons. We found that the structural features of the glycine-binding sites in both GluN1 and GluN3A subunits are correlated with receptor forward trafficking to the cell surface. In addition, we found that a potentially clinically relevant mutation in the glycine-binding site of the human GluN3A subunit significantly reduces surface delivery of NMDARs. Taken together, these findings provide novel insight into how NMDARs are regulated by their glycine-binding sites and may provide important information regarding the role of NMDARs in both physiological and pathophysiological processes in the mammalian CNS.

## Introduction

*N*-methyl-D-aspartate receptors (NMDARs) are a subclass of glutamate receptors that play an essential role in synapse development, excitatory neurotransmission as well as synaptic plasticity in the mammalian central nervous system (CNS)^1,2^. It has been well established that dysregulation of NMDARs plays a critical role in the aetiology of many neuropsychiatric and neurological disorders and conditions, including Huntington’s disease^3,4^, schizophrenia^5^, cocaine addiction^6^, and nicotine dependence^7^. In addition, a growing number of studies suggest that many neuropsychiatric disorders are associated with mutations in genes that encode various NMDAR subunits, including the GluN1^8,9^, and GluN3A^10,11^ subunits. Thus, understanding the molecular mechanisms that regulate NMDARs is an essential step towards designing effective therapies for these patients.

Structurally, NMDARs are heterotetramers composed of GluN1 (with eight splice variants), GluN2 (GluN2A through GluN2D), and/or GluN3 (GluN3A and GluN3B) subunits^2,12^. All GluN subunits have a common membrane topology, including an extracellular amino-terminal domain (ATD), extracellular ligand-binding domains (LBDs) formed by S1 and S2 segments, four membrane domains (M1 through M4), and an intracellular C-terminal domain (CTD)^2,12^. The conventional NMDAR subtype ‒ GluN1/GluN2 ‒ is activated by the binding of agonists to the glutamate-binding site in the LBD of GluN2 together with the simultaneous binding of a co-agonist to the glycine-binding site in the LBD of GluN1^2,13–15^. Interestingly, unconventional NMDAR subtypes ‒ namely, GluN1/GluN3A and GluN1/GluN3B receptors ‒ are activated by the binding of agonist to the glycine-binding site in the LBD of the GluN3 subunit, whereas binding of a co-agonist to the glycine-binding site in the LBD of GluN1 drives the desensitisation of glycine-induced currents in GluN1/GluN3 receptors^16–19^. Thus, the glycine-binding sites in the LBD of various GluN subunits play distinct functional roles in NMDARs.

Both the number and type of NMDARs present at the neuronal surface are regulated at multiple levels^20–22^, including their synthesis^23,24^, subunit assembly^25–28^, processing within the endoplasmic reticulum (ER)^29–36^, trafficking to the cell membrane^37–39^, lateral diffusion^40,41^, internalisation/recycling^42–45^, and degradation^45,46^. The ER quality control retains unassembled GluN1 (except the GluN1-2, GluN1-3, and GluN1-4 splice variants)^31–33^, GluN2^34,35^, and GluN3 subunits^30,36^. This is supported by experiments in mice that lack the GluN1 subunit in the hippocampus, in which GluN2 subunits accumulate in the ER^47^. In addition, a wide variety of regions in GluN subunits ‒ including the LBD ‒ are critical for regulating the surface delivery of NMDARs^48,49^. Specifically, Kenny *et al*. previously reported that disrupting the glycine-binding site in the LBD of GluN1 by introducing the D732A mutation reduces the surface delivery of GluN1/GluN2A receptors^49^. Similarly, She *et al*. reported that the glutamate-binding site in the LBD of GluN2B regulates the surface delivery of GluN1/GluN2B receptors^48^; this finding was supported by a recent study using human GluN1/GluN2B receptors with known pathogenic mutations^50^. However, whether structural changes in the glycine-binding sites in the GluN1 and/or GluN3A subunits regulate the surface delivery of functional GluN3A-containing NMDARs is currently unknown.

Here, we used a combination of microscopy, quantitative assays and electrophysiology in mammalian cell lines and primary rat hippocampal neurones in order to investigate whether disrupting the structure of the glycine-binding sites in the LBDs of GluN1 and/or GluN3A subunits affects the surface delivery of GluN3A-containing NMDARs. We found that mutant GluN1 and GluN3A subunits have reduced glycine sensitivity, and this reduction is correlated with reduced forward trafficking of NMDARs to the cell surface. These results were supported by additional experiments using the human GluN3A subunit. Taken together, our results provide novel insight into the mechanisms that regulate the number, type, and function of NMDARs at the neuronal surface.

## Results

### The S1 segment of the LBD in GluN3A subunit is essential for the surface delivery of GluN1/GluN3A receptors

Previous studies revealed that different domains in GluN1 and GluN2 regulate the surface expression of NMDARs^2,20^. We therefore examined whether the N-terminal and/or C-terminal regions of GluN3A subunits regulate the surface expression of NMDARs by expressing wild-type and mutant GluN1 and GluN3A subunits in COS-7 cells (a cell line derived from African green monkey kidney fibroblast; acronym “COS” is derived from the cells being CV-1 (simian) in Origin, and carrying the SV40 genetic material) and Human Embryonic Kidney 293 (HEK293) cells (which do not express endogenous GluN subunits), and in cultured hippocampal neurones. Our group^51^ and others^52,53^ previously reported that GluN1-4a/GluN3A receptors produce larger glycine-induced currents compared to GluN1-1a/GluN3A receptors; therefore, we used the GluN1-4a subunit in our experiments with COS-7 and HEK293 cells.

**Figure 1:**
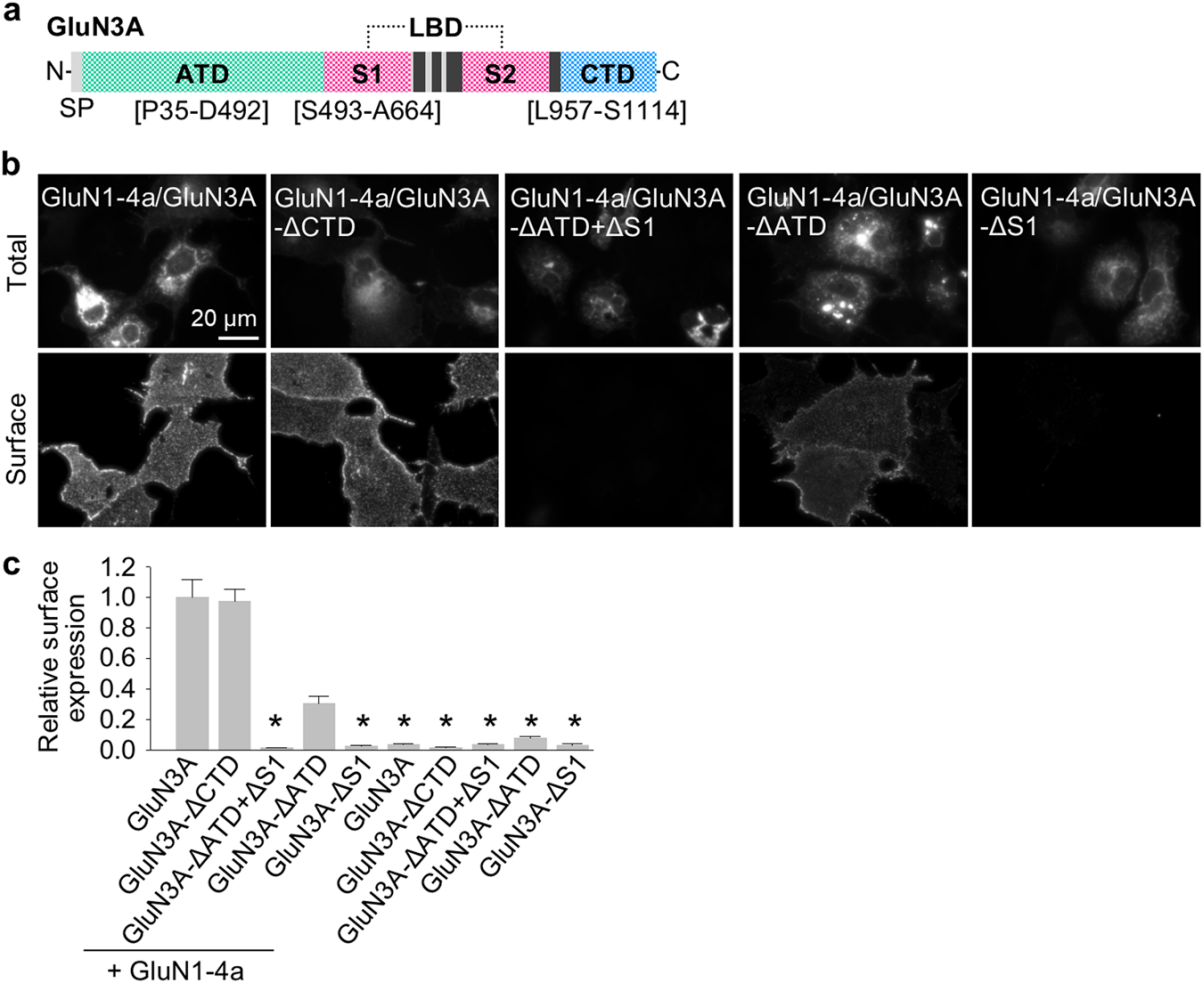
The S1 segment in the GluN3A subunit is essential for the surface expression of GluN1/GluN3A receptors. **(a)** Schematic diagram of the GluN3A subunit, with the amino-terminal domain (ATD), ligand-binding domain (LBD) composed of S1 and S2 segments, and C-terminal domain (CTD) indicated; the black rectangles indicate membrane domains. **(b)** Representative images of total and surface GluN1-4a/GFP-GluN3A receptors lacking the indicated domains in the GluN3A subunit expressed in COS-7 cells and labelled 24 h after transfection. **(c)** Summary of the relative surface expression of the indicated GluN subunits measured using fluorescence microscopy (n ≥ 20 cells per group); *p < 0.05 vs. GluN1-4a/GluN3A (ANOVA).

We first generated rat GluN3A subunits that lack the entire CTD (GluN3A-ΔCTD) or the entire ATD and S1 segment (GluN3A-ΔATD + ΔS1; Fig. 1a) and expressed each GluN3A subunit in COS-7 cells either alone or together with GluN1-4a subunit (Fig. 1b). We found that GluN1-4a/GluN3A-ΔCTD receptors are delivered to the surface at wild-type levels; in contrast, GluN1-4a/GluN3A-ΔATD + ΔS1 receptors failed to traffic to the cell surface (Fig. 1b,c). Next, we examined receptors containing GluN3A subunits that lack either the ATD (GluN3A-ΔATD) or the S1 segment (GluN3A-ΔS1; Fig. 1a) ‒ and found that GluN1-4a/GluN3A-ΔATD receptors are expressed at the cell surface (albeit at reduced levels compared to wild-type GluN1-4a/GluN3A receptors), whereas GluN1-4a/GluN3A-ΔS1 receptors failed to traffic to the cell surface (Fig. 1b,c; see also the Discussion). Consistent with our above-mentioned findings, when expressed alone (i.e. without GluN1) the truncated GluN3A subunits failed to reach the cell surface (Fig. 1c). The total expression levels were not significantly different among the studied GluN subunit combinations (Supplementary Fig. S1). These findings indicate that the S1 segment of the GluN3A subunit is required for the surface delivery of GluN1/GluN3A receptors.

### The glycine-binding sites in both GluN1 and GluN3A subunits regulate the number of NMDARs at the cell surface and their function in both cell lines and hippocampal neurones

Previous studies showed that the surface delivery of GluN1/GluN2 receptors is regulated by the glycine-binding site in the GluN1 subunit^49^ and the glutamate-binding site in the GluN2 subunit^48^. Here, we examined whether the glycine-binding sites in the GluN1 or GluN3A subunits also regulate the surface delivery of GluN3A-containing NMDARs.

**Figure 2:**
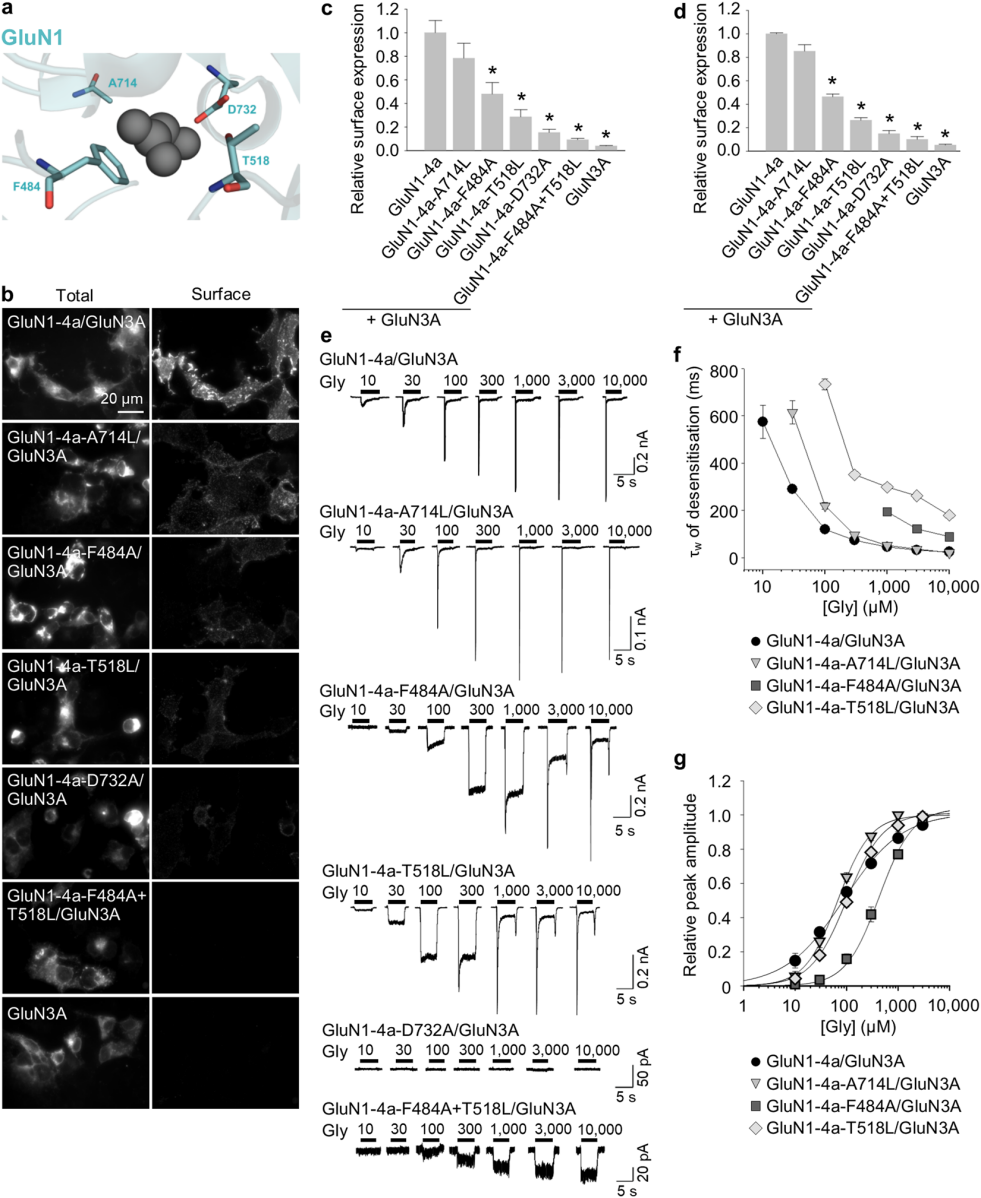
Mutations in the glycine-binding site in the GluN1 subunit alter the surface expression and desensitisation kinetics of GluN1/GluN3A receptors. **(a)** Schematic representation of the glycine-binding site in the GluN1 subunit (PDB code: 1PB7); the amino acid residues studied here, and a glycine molecule, are shown. (**b**) Representative images of COS-7 cells transfected with the indicated wild-type or mutant GluN1-4a subunits together with GFP-GluN3A (GluN3A) subunit and labelled 24 h after transfection. **(c)** Summary of the relative surface expression of the indicated GluN subunits measured using fluorescence microscopy (n ≥ 24 cells per group); *p < 0.05 vs. GluN1-4a/GluN3A (ANOVA). **(d)** Quantification of relative surface expression of the indicated GluN1-4a/GFP-GluN3A (GluN1-4a/GluN3A) receptors using quantitative colorimetric assay is shown (n = 6); *p < 0.05 relative to GluN1-4a/GluN3A (ANOVA). (**e**) Representative whole-cell patch-clamp recordings from HEK293 cells transfected with the indicated wild-type or mutant GluN1-4a/GluN3A receptors. Currents were elicited by applying glycine at the indicated concentrations. (**f**) Summary of the τ_w_ of desensitisation for the indicated GluN1-4a/GluN3A receptors (n ≥ 5 cells per group). (**g**) Peak concentration-response curves for the indicated GluN1-4a/GluN3A receptors. Each data point represents the mean relative current from ≥5 independent cells. The EC_50_ values and Hill coefficients are listed in Table 1.

We first used mutations in the GluN1 subunit that were shown previously to alter the EC_50_ for glycine binding to GluN1/GluN2 receptors^17,54^ (Fig. 2a). Specifically, we introduced the A714L mutation in GluN1-4a subunit, which stabilises the open conformation of the LBD and slightly reduces the receptor’s sensitivity to glycine; other mutations in GluN1-4a subunit included F484A, T518L^17^, and D732A^49,54^, all of which reduce glycine affinity by two orders of magnitude. In addition, we used the double mutant GluN1-4a-F484A + T518L subunit, which was previously reported to be glycine-insensitive at concentrations up to 30 mM when co-expressed with the GluN2A subunit^17^.

First, we co-expressed each mutant GluN1-4a subunit together with GluN3A subunit in COS-7 cells and measured surface expression using fluorescence microscopy (Fig. 2b,c) and in HEK293 cells using quantitative assay of surface expression (Fig. 2d). The total expression levels were not significantly different among the studied GluN subunit combinations (Supplementary Fig. S1). We found that the surface expression was highest for GluN1-4a/GluN3A receptors, followed by (in decreasing order of surface expression) GluN1-4a-A714L/GluN3A, GluN1-4a-F484A/GluN3A, GluN1-4a-T518L/GluN3A, GluN1-4a-D732A/GluN3A, and GluN1-4a-F484A + T518L/GluN3A receptors; interestingly, this rank order corresponds to the previously determined order of glycine EC_50_ values for GluN1/GluN2 receptors containing these mutant GluN1 subunits (Supplementary Fig. S2).

Because the previously assessed EC_50_ values for glycine were determined using GluN1/GluN2 receptors expressed in *Xenopus* oocytes, they might not necessarily reflect the change in glycine affinity of GluN1/GluN3A receptors expressed in mammalian cells. We therefore measured the whole-cell currents induced by increasing concentrations of glycine (ranging from 10 µM to 10 mM) in HEK293 cells co-expressing GluN1/GluN3A subunits (Fig. 2e); importantly, using a rapid solution exchange system enabled us to detect the peak glycine-induced current prior to receptor desensitisation. In these experiments, we transfected HEK293 cells using different amounts of GluN cDNAs, as some GluN1-4a/GluN3A combinations otherwise yielded small glycine-induced currents (the following ratios of cDNAs encoding the GluN1-4a and GluN3A subunits were used: GluN1-4a/GluN3A (1:1), GluN1-4a-A714L/GluN3A (1:1), and GluN1-4a-F484A/GluN3A (1:1); GluN1-4a-T518L/GluN3A (2:1), GluN1-4a-D732A/GluN3A (2:1), and GluN1-4a-F484A + T518L/GluN3A (2:1)); therefore, we were unable to directly compare the absolute peak current amplitudes among the various GluN1-4a/GluN3A receptors. Nevertheless, we found that the time constant of desensitisation (τ_w_) differed among the various mutant receptors tested and corresponded with the same rank order that we observed for surface delivery (Fig. 2f and Supplementary Fig. S2). Maximum steady-state currents carried by wild-type GluN1-4a/GluN3A receptors activated by 1 mM glycine were in range of 5 to 25 pA (n = 10) which precluded reliable concentration-response analysis of steady-state currents. Our analysis of the peak concentration-response relationship revealed that only the GluN1-4a-F484A mutation caused a rightward shift of the curve (Fig. 2g), resulting in an ~5-fold decrease in glycine potency compared to wild-type GluN1-4a/GluN3A receptors (Table 1); this finding is consistent with previous reports that activation of GluN1/GluN3A receptors is regulated primarily by the glycine-binding site in GluN3A subunit^16–18^. In contrast, the Hill coefficient (*h*) was increased for all receptors containing mutant GluN1-4a subunits compared to wild-type receptors (Table 1), which indicates that the mutations change the degree of cooperativity between the glycine-binding sites in the GluN1 subunits^55^. In contrast to our results described above, the determined EC_50_ values for the mutant GluN1-4a/GluN3A receptors tested did not correlate with their surface expression levels (Supplementary Fig. S2). Cells expressing GluN1-4a-D732A/GluN3A receptors had no detectable currents, even at the highest glycine concentration tested; moreover, at all glycine concentrations tested, currents measured through GluN1-4a-F484A + T518L/GluN3A receptors did not desensitise (Fig. 2e). Thus, we were unable to perform detailed functional analyses of these two mutant receptors (see also the Discussion). Taken together, these data support the hypothesis that structural changes in the glycine-binding site in the GluN1 subunit are associated with changes in the surface delivery and the desensitisation properties of GluN1/GluN3A receptors.

**Table 1:** Summary of the fitting parameters for the peak concentration-response relationship measured for wild-type and mutant GluN1/GluN3A receptors expressed in HEK293 cells (see Figs 2g and 3g).

Receptor	EC_50_ (μM)^a^	*h* ^a^	n
GluN1-4a/GluN3A	89.0 ± 3.1	0.77 ± 0.04	6
GluN1-4a-A714L/GluN3A	67.6 ± 7.5	1.37 ± 0.11*	7
GluN1-4a-T518L/GluN3A	101.6 ± 17.4	1.25 ± 0.12*	6
GluN1-4a-F484A/GluN3A	429.3 ± 48.4*	1.34 ± 0.05*	8
GluN1-4a/GluN3A-T825L	785.0 ± 189.0*	1.82 ± 0.35*	5
GluN1-4a/GluN3A-Y605A	2566.6 ± 385.9*	1.17 ± 0.17	6

^a^The EC_50_ values and Hill coefficient (h) were obtained by fitting the normalized data from each cell using Equation (1). Data are presented as the mean ± SEM, and n corresponds to the number of cells recorded. Statistical analysis was performed for logEC_50_ and logHill values; *p < 0.05 vs. GluN1-4a/GluN3A (one-way ANOVA with post-hoc Dunnett’s test).

**Figure 3:**
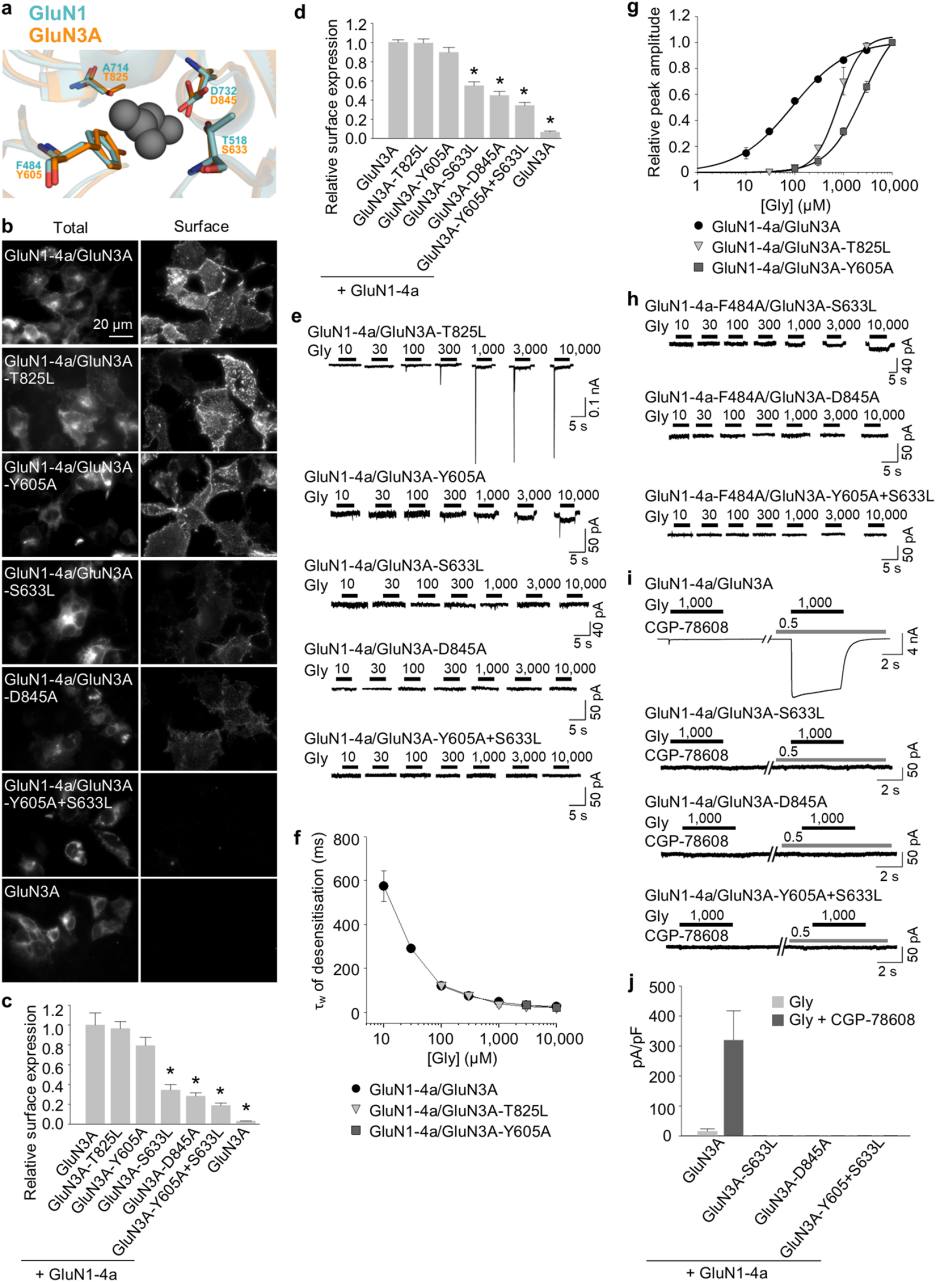
Mutations in the glycine-binding site in the GluN3A subunit regulate the surface expression and glycine affinity of GluN1/GluN3A receptors. (**a**) Schematic representation of the glycine-binding site in the GluN3A subunit (PDB code: 2RC7; in orange), with a glycine molecule shown; for comparison, the binding site in the GluN1 subunit is shown in blue. (**b**) Representative images of COS-7 cells transfected with the indicated wild-type or mutant GFP-GluN3A subunit together with GluN1-4a subunit and labelled 24 h after transfection. **(c)** Summary of the relative surface expression of the indicated GluN subunits measured using fluorescence microscopy (n ≥ 20 cells per group); *p < 0.05 vs. GluN1-4a/GluN3A (ANOVA). (**d**) Heterologous HEK293 cells co-expressing the indicated GluN1-4a and GFP-GluN3A (GluN3A) subunits were labeled with primary anti-GFP and secondary antibodies in non-permeabilizing and permeabilizing conditions. The bar graphs show quantification of relative surface expression of the indicated GluN subunit combinations obtained using quantitative colorimetric assay (n = 6); *p < 0.05 relative to GluN1-4a/GluN3A (ANOVA). (**e,h**) Representative whole-cell patch-clamp recordings from HEK293 cells transfected with the indicated wild-type or mutant GluN1-4a/GluN3A receptors. Currents were elicited by applying the indicated concentrations of glycine. (**f**) Summary of the τ_w_ of desensitisation for the indicated GluN1-4a/GluN3A (n ≥ 5 cells per group). (**g**) Peak concentration-response curves for the indicated wild-type and mutant GluN1-4a/GluN3A receptors. Each data point represents the mean relative current from ≥5 cells. The EC_50_ values and Hill coefficients are listed in Table 1. (**i**) The pharmacological analysis with CGP-78608 at the GluN1/GluN3A receptors. Representative whole-cell patch-clamp recordings from HEK293 cells transfected with the indicated wild-type or mutant GluN1-4a/GluN3A receptors at a membrane potential of −60 mV. Currents were elicited by applying 1,000 µM glycine; 0.5 µM CGP78608 was applied as indicated. (**j**) Summary of current densities (pA/pF) obtained from the HEK293 cells expressing the indicated GluN1-4a/GluN3A receptors (n ≥ 6 cells per group).

Given that the glycine-binding sites in the LBD of GluN1 and GluN3A subunits are highly conserved, we next asked whether similar structural changes in the glycine-binding site in the GluN3A subunit can also affect the surface delivery of GluN1/GluN3A receptors. Accordingly, we introduced point mutations in GluN3A subunit at the positions analogous to GluN1 subunit, yielding four mutant GluN3 subunits (GluN3A-T825L, GluN3A-Y605A, GluN3A-S633L, and GluN3A-D845A) and the double mutant GluN3A-Y605A + S633L subunit (Fig. 3a); each subunit was then co-expressed together with GluN1-4a subunit in COS-7 cells and surface expression was examined using microscopy (Fig. 3b,c) and in HEK293 cells using quantitative assay of surface expression (Fig. 3d). The total expression levels were not significantly different among the studied GluN subunit combinations (Supplementary Fig. S1). Interestingly, the rank order of surface expression for the receptors containing mutant GluN3A subunits was similar to the order that we observed with the analogous mutant GluN1 subunits (Supplementary Fig. S2). Specifically, receptors containing the GluN3A-T825L subunit had the highest surface expression, followed by (in decreasing order) GluN3A-Y605A, GluN3A-S633L, GluN3A-D845A, and GluN3A-Y605A + S633L subunits.

Next, we measured the functional properties of GluN1-4a/GluN3A receptors containing wild-type or mutant GluN3A subunits expressed in HEK293 cells in order to examine the effect of these mutations on glycine affinity. We found that both GluN1-4a/GluN3A-T825L and GluN1-4a/GluN3A-Y605A receptors produced detectable currents (1:2 ratios of cDNAs encoding the GluN1-4a and GluN3A subunits were used), whereas the other three mutant receptors failed to produce measurable currents, even with 10 mM glycine (Fig. 3e). Moreover, these two mutant receptors had a τ_w_ of desensitisation similar to wild-type GluN1-4a/GluN3A receptors (Fig. 3f), which is consistent with the notion that the desensitisation of GluN1/GluN3A receptors is regulated by the glycine-binding site in the GluN1 subunit. An analysis of the peak concentration-response relationship revealed that glycine is 9-fold and 29-fold less potent at activating GluN1-4a/GluN3A-T825L and GluN1-4a/GluN3A-Y605A receptors, respectively, compared to wild-type GluN1-4a/GluN3A receptors (Fig. 3g and Table 1), which clearly correlates with their surface expression levels (Supplementary Fig. S2). In addition, the decreased glycine potency in GluN1-4a/GluN3A-T825L receptors was accompanied by an increase in the Hill coefficient (Table 1), suggesting a change in the degree of cooperativity between the glycine-binding sites in the GluN3A subunits. These data support the hypothesis that activation of GluN1/GluN3A receptors is mediated by glycine binding to the GluN3A subunit.

As noted above, we measured no detectable currents in cells expressing GluN1-4a/GluN3A-S633L, GluN1-4a/GluN3A-D845A, or GluN1-4a/GluN3A-Y605A + S633L receptors (Fig. 3e). This finding may be explained by the greatly reduced number of receptors at the cell surface, a loss of glycine binding, and/or a change in the desensitisation properties of these mutant receptors. To address these possibilities, we co-expressed GluN3A-S633L, GluN3A-D845A, and GluN3A-Y605A-S633L subunits together with the mutant GluN1-4a-F484A subunit, which slows the receptor’s desensitisation kinetics (Fig. 2f) and does not prevent surface delivery of the GluN1/GluN3A receptors (Fig. 2). We found that cells expressing GluN1-4a-F484A/GluN3A-S633L receptors had detectable currents when glycine was applied at 1, 3, or 10 mM, whereas no currents were detected in cells expressing GluN1-4a-F484A/GluN3A-D845A or GluN1-4a-F484A/GluN3A-Y605A + S633L receptors, even with 10 mM glycine (Fig. 3h). In addition, we performed electrophysiological recordings with compound CGP-78608 using HEK293 cells expressing GluN3A, GluN3A-S633L, GluN3A-D845A, and GluN3A-Y605A-S633L subunits together with the GluN1-4a subunit. In the agreement with recent study^56^, we observed profound potentiating effect of CGP-78608 at the wild-type GluN1-4a/GluN3A receptors exposed to 1 mM (Fig. 3i,j) or 10 mM (Supplementary Fig. S3) glycine. However, by using the CGP-78608 with 1 mM glycine (Fig. 3i,j) or even with 10 mM glycine (Supplementary Fig. S3) we were not able to observe responses from the HEK293 cells expressing the GluN1-4a/GluN3A-S633L, GluN1-4a/GluN3A-D845A and GluN1-4a/GluN3A-Y605A + S633L receptors. Together, our data suggest that the glycine-binding sites in both the GluN1 and GluN3A subunits regulate the surface delivery of GluN1/GluN3A receptors in the mammalian cell lines.

**Figure 4:**
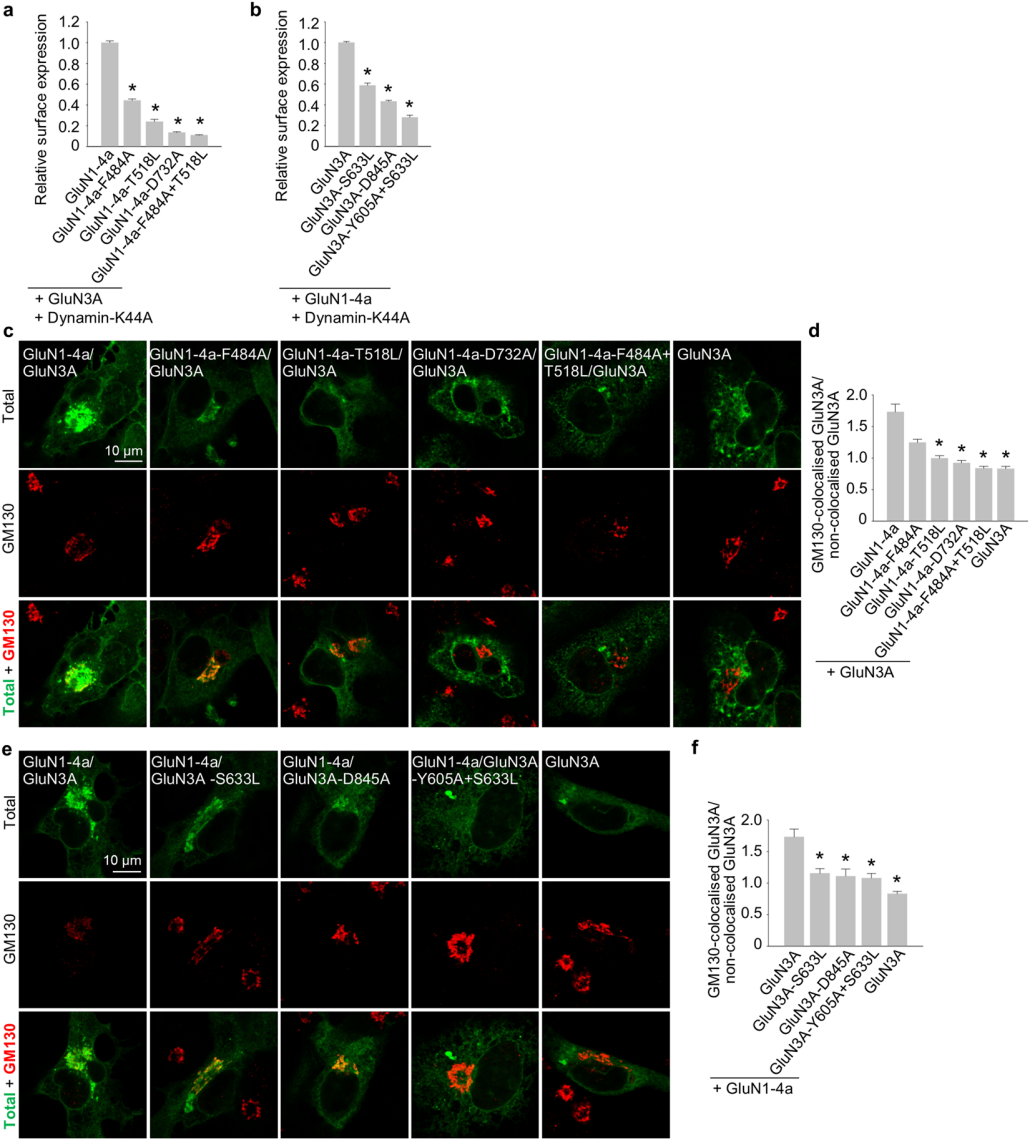
The glycine-binding sites in the GluN1 and GluN3A subunits regulate forward trafficking of GluN1/GluN3A receptors (**a,b**) HEK293 cells co-expressing the indicated GluN1-4a and GFP-GluN3A (GluN3A) subunits together with the dynamin-K44A were labelled with primary anti-GFP and secondary antibodies in non-permeabilizing and permeabilizing conditions. The bar graphs show quantification of relative surface expression of the indicated GluN subunit combinations obtained using quantitative colorimetric assay (n = 6); *p < 0.05 relative to GluN1-4a/GluN3A (ANOVA). (**c,e**) Representative confocal microscopy images of the COS-7 cells transfected with the indicated GluN1-4a and GFP-GluN3A (GluN3A) subunits; the anti-GM130 antibody was used to label the GA. (d,f) Summary of the average intensity of GFP-GluN3A (GluN3A) subunit signal colocalised with GM130 over the average intensity of GFP-GluN3A (GluN3A) subunit signal outside GM130 signal, calculated for the indicated GluN subunit combinations (n ≥ 10 cells); *p < 0.05 vs. GluN1-4a/GluN3A; ANOVA).

In order to separate the relative contributions of forward trafficking from endocytosis, we employed a mutated version of K44A HA-dynamin 2 (dynamin-K44A) which inhibits clathrin-dependent endocytosis, using similar approach as reported previously for the mutated GluN1/GluN2 receptors^48^. We performed the quantitative assays on the HEK293 cells expressing the GluN1/GluN3A receptor combinations which exhibited significantly reduced surface expression (Figs 2d and 3d), in the presence of dynamin-K44A. In these experiments, we did not observe altered pattern of surface delivery or total expression of the respective mutated GluN1/GluN3A receptors (Fig. 4a,b and Supplementary Fig. S1; compare with Figs 2d and 3d and Supplementary Fig. S1), indicating that glycine binding regulates forward trafficking rather than internalization of GluN1/GluN3A receptors. To further corroborate this conclusion, we next labelled the COS-7 cells expressing the mutated GluN1/GluN3A receptors which exhibited reduced surface delivery in the experiments described above, with a Golgi apparatus (GA) marker Golgi matrix protein 130 (GM130; Fig. 4c,e). Our microscopical analysis revealed significantly decreased colocalisation of the GluN1-4a-T518L/GluN3A, GluN1-4a-D732A/GluN3A and GluN1-4a-F484A + T518L/GluN3A and GluN3A subunit combinations with the GM130 when compared with the wild-type GluN1-4a/GluN3A receptors (Fig. 4d). Concerning the GluN1-4a-F484A/GluN3A receptors, we did not observe significant tendency for their reduced colocalisation with the GM130, which resembles previously reported finding with the GluN1/GluN2B-S664G receptors^48^. Our microscopical analysis further revealed decreased colocalisation of the GluN1-4a/GluN3A-S633L, GluN1-4a/GluN3A-D845A and GluN1-4a/GluN3A-Y605A + S633L receptors with the GM130 when compared with the wild-type GluN1-4a/GluN3A receptors (Fig. 4f). Together, these data indicate that impaired glycine binding reduces forward trafficking of GluN1/GluN3A receptors to the cell surface.

**Figure 5:**
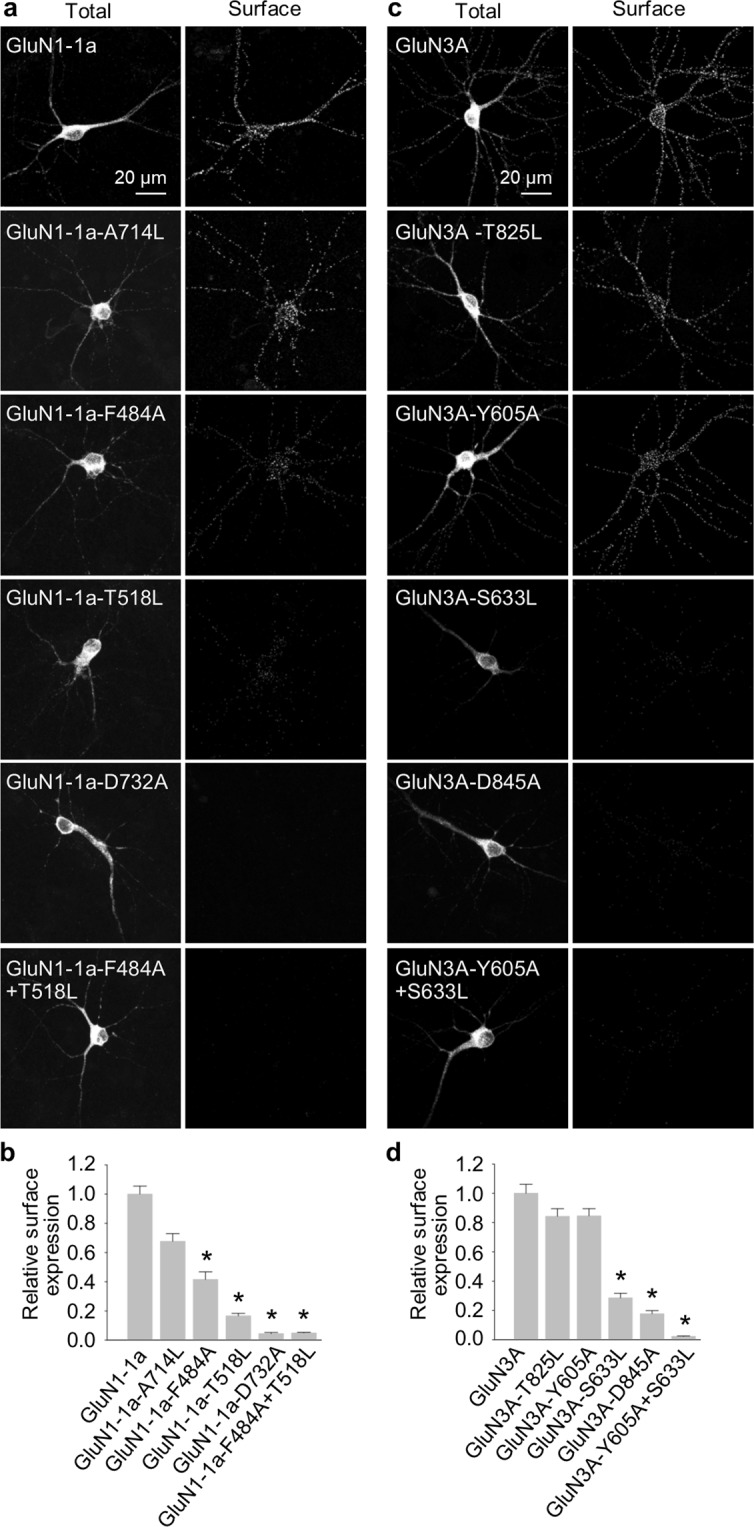
The glycine-binding sites in the GluN1 and GluN3A subunits regulate the surface expression of NMDARs in hippocampal neurones. (**a,c**) Representative images of cultured rat hippocampal neurones transfected at DIV10 with the indicated YFP-GluN1-1a **(a)** or GFP-GluN3A **(c)** subunits; at DIV14, surface and total subunits were stained using an anti-GFP antibody. (**b,d**) Summary of the relative surface expression of the indicated subunits measured in 10-µm segments of secondary or tertiary dendrites (n ≥ 30 segments in ≥ 6 different cells per group); *p < 0.05 vs. GluN1-1a **(b)** or GluN3A **(d**) (ANOVA).

Next, we asked whether the introducing of mutations in the glycine-binding sites of GluN subunits also affects the trafficking of NMDARs in neurones. We therefore transfected cultured rat hippocampal neurones at DIV10 with YFP-tagged wild-type or mutant GluN1-1a subunits (we chose this splice variant because it is not delivered to the cell surface unless associated with the GluN2 and/or GluN3 subunit) or GFP-tagged wild-type or mutant GluN3A subunits. Four days after transfection (i.e. at DIV14), we then measured the surface and total pools of recombinant GluN subunits using labelling with an anti-GFP antibody and confocal microscopy. Our analysis revealed the following rank order for the surface delivery of mutant GluN1-1a subunits (in decreasing order): GluN1-1a-A714L, GluN1-1a-F484A, GluN1-1a-T518L, GluN1-1a-D732A, and GluN1-1a-F484A + T518L (Fig. 5a,b). Similarly, all five mutant GluN3 subunits exhibited the following rank order of surface delivery (in decreasing order): GluN3A-T825L, GluN3A-Y605A, GluN3A-S633L, GluN3A-D845A, and GluN3A-Y605A + S633L (Fig. 5c,d). The total expression levels were not significantly different among the studied GluN subunits (Supplementary Fig. S1). These results are consistent with our cell surface expression data in cell lines and support the notion that the glycine-binding sites in both the GluN1 and GluN3A subunits are critical for surface delivery of NMDARs.

### Clinically relevant mutation in the glycine-binding site of GluN3A subunit alters the surface delivery of NMDARs

**Figure 6:**
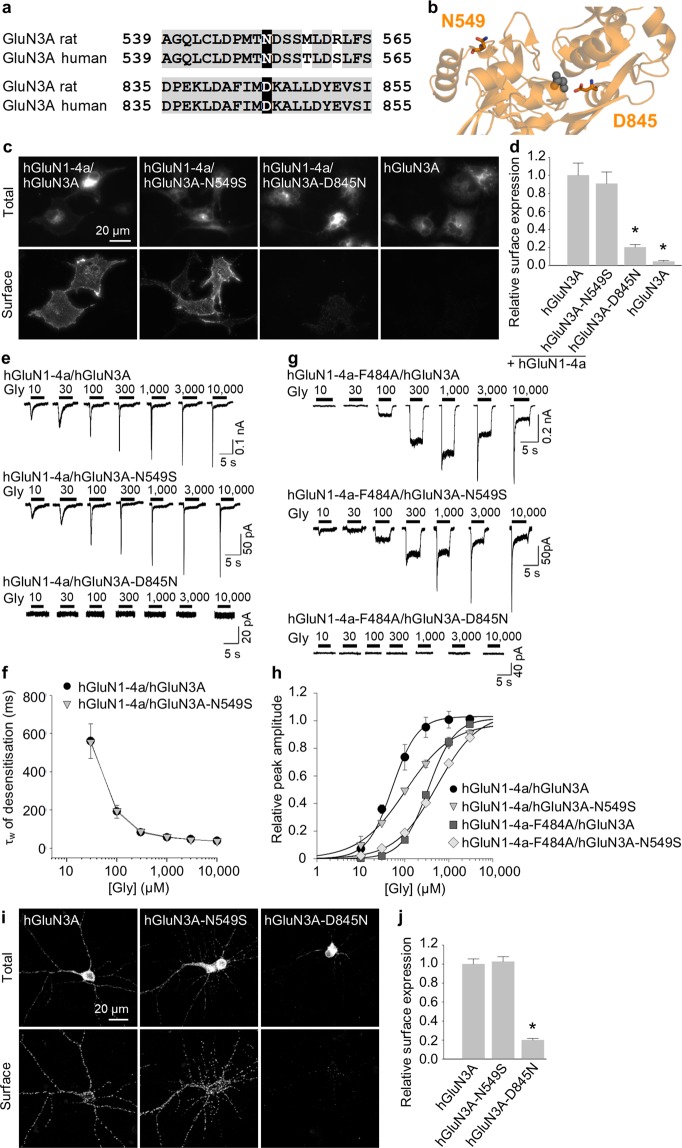
Clinically relevant mutation in the LBD of the GluN3A subunit alters the surface delivery of NMDARs. (**a**) Sequence alignment of the rat and human GluN3A subunits, with two clinically relevant mutations in the LBD shown in black rectangles. The numbers refer to the amino acid residues. **(b)** Schematic representation of the part of the LBD of the GluN3A subunit (PDB code: 2RC7); the amino acid residues studied here, and a glycine molecule, are shown. **(c)** Representative images of COS-7 cells transfected with the indicated human GFP-GluN3A (hGluN3A) and GluN1-4a (hGluN1-4a) subunits. **(d)** Summary of the relative surface expression of the indicated hGluN1-4a/hGluN3A subunits measured using fluorescence microscopy (n ≥ 18 cells per group); *p < 0.05 vs. hGluN1-4a/hGluN3A (ANOVA). **(e,g)** Examples of whole-cell patch-clamp recordings from HEK293 cells transfected with the indicated hGluN1-4a/hGluN3A receptors. Currents were elicited by applying the indicated concentration of glycine. **(f)** Summary of the τ_w_ of desensitisation for the indicated hGluN1-4a/hGluN3A receptors (n ≥ 5 cells per group). **(h)** Peak concentration-response curves for the indicated wild-type and mutant hGluN1-4a/hGluN3A receptors. Each data point represents the mean relative currents recorded from ≥4 cells. The EC_50_ values and Hill coefficients are listed in Table 2. **(i)** Representative images of hippocampal neurones transfected at DIV10 with wild-type or mutant hGluN3A subunits and labelled at DIV14. **(j)** Summary of the relative surface expression of the indicated subunits measured in 10-µm segments of secondary or tertiary dendrites (n ≥ 50 segments from ≥10 different cells per group); *p < 0.05 vs. hGluN3A (ANOVA).

A growing body of data indicates that many neuropsychiatric disorders are associated with mutations in genes that encode NMDAR subunits, including the GluN3A subunit^10,11^; however, in most cases how these mutations affect the trafficking and/or function of NMDARs is unknown. To address this question, we performed a database search and identified two clinically relevant mutations in the human GluN3A (hGluN3A) subunit; both of these residues are conserved between rat and human GluN3A subunits (Fig. 6a). The N549S mutation is located within the LBD but is likely not involved in forming of the glycine-binding site, whereas the D845N mutation is present within the glycine-binding site (Fig. 6b; see the Discussion for further information).

We first generated the human GluN1-4a subunit (hGluN1-4a) as well as GFP-tagged wild-type hGluN3A, and mutated hGluN3A-N549S, and hGluN3A-D845N subunits. We then co-expressed these hGluN1-4a and hGluN3A subunits in COS-7 cells and measured surface expression of the receptors containing either wild-type or mutant hGluN3A subunits using an anti-GFP antibody and fluorescent microscopy. We found that although hGluN1-4a/hGluN3A-N549S receptors were expressed at the cell surface at the same level as wild-type hGluN1-4a/hGluN3A receptors; the surface expression of hGluN1-4a/hGluN3A-D845N receptors was significantly reduced (Fig. 6c,d). The total expression levels were not significantly different among the studied hGluN subunit combinations (Supplementary Fig. S1).

Next, we measured the functional properties of mutant hGluN3A subunits co-expressed in HEK293 cells together with hGluN1-4a subunit (1:2 ratios of cDNAs encoding the hGluN1-4a and hGluN3A subunits were used). Consistent with robust expression at the cell surface, hGluN1-4a/hGluN3A-N549S receptors produced glycine-induced currents at all concentrations tested (Fig. 6e), with a τ_w_ of desensitisation similar to wild-type hGluN1-4a/hGluN3A receptors (Fig. 6f). In contrast ‒ and consistent with our surface delivery findings obtained with COS-7 cells ‒ no detectable glycine-induced currents were measured in HEK293 cells expressing hGluN1-4a/hGluN3A-D845N receptors, even at the highest glycine concentration tested (Fig. 6e). We therefore introduced the F484A mutation into the hGluN1-4a subunit and co-expressed this mutant hGluN1-F484A subunit together with wild-type or mutant hGluN3A subunits in HEK293 cells. As expected, both hGluN1-4a-F484A/hGluN3A and hGluN1-4a-F484A/hGluN3A-N549S receptors produced glycine-induced currents with reduced desensitisation kinetics (Fig. 6g). However, no detectable glycine-induced currents were measured in cells expressing hGluN1-4a-F484A/hGluN3A-D845N receptors (Fig. 6g), supporting our previous finding that the D845N mutation drastically reduces the surface delivery of hGluN1/hGluN3A receptors. Our analysis of the peak concentration-response relationship for wild-type and mutant hGluN1-4a/hGluN3A receptors revealed that glycine is ~2-fold less potent at activating both hGluN1-4a/hGluN3A-N549S and hGluN1-4a-F484A/hGluN3A-N549S receptors compared to their respective wild-type receptors (Fig. 6h and Table 2). The reduced glycine potency in both the hGluN1-4a/hGluN3A-N549S and hGluN1-4a-F484A/hGluN3A-N549S receptors was accompanied by a decrease in the Hill coefficient (Table 2), consistent with the altered cooperativity between the glycine-binding sites in the hGluN3A subunits.

**Table 2:** Summary of the fitting parameters for the peak concentration-response relationship measured for wild-type and mutant hGluN1/hGluN3A receptors expressed in HEK293 cells (see Fig. 6h).

Receptor	EC_50_ (μM)^a^	*h* ^a^	n
hGluN1-4a/hGluN3A	52.0 ± 8.2	1.52 ± 0.23	4
hGluN1-4a/hGluN3A-N549S	103.0 ± 7.9*	0.82 ± 0.04*	5
hGluN1-4a-F484A/hGluN3A	342.2 ± 24.4	1.45 ± 0.07	9
hGluN1-4a-F484A/hGluN3A-N549S	536.4 ± 53.9*	0.92 ± 0.05*	8

^a^The EC_50_ values and Hill coefficient (h) were obtained by fitting the normalized data from each cell using Equation (1). Data are presented as the mean ± SEM, and n corresponds to the number of cells recorded. Statistical analysis was performed for logEC_50_ and logHill values; *p < 0.05 vs. hGluN1-4a/hGluN3A or hGluN1-4a-F484A/hGluN3A (Student’s t-test).

Next, we measured the surface expression of wild-type and mutant hGluN3A subunits expressed in cultured hippocampal neurones, using the same strategy we used above with rat GluN subunits (see Fig. 5). Consistent with our data from COS-7 and HEK293 cells, we found that the hGluN3A-N549S subunit was delivered to the neuronal cell surface at the same level as the wild-type hGluN3A subunit, whereas the surface expression of the hGluN3A-D845N subunit was significantly reduced (Fig. 6i,j). The total expression levels were not significantly different among the studied hGluN3A subunits (Supplementary Fig. S1). Taken together, these data provide compelling evidence that the glycine-binding site in the GluN3A subunit plays a critical role in the surface delivery of both rat and human NMDARs.

## Discussion

The relatively long N- and C-termini of GluN subunits is believed to allow the receptor to interact with a variety of proteins during its journey to the cell surface, at the cell surface, and during internalisation (for more details see Introduction section). We found that GluN1/GluN3A receptors with a truncated CTD in the GluN3A subunit are delivered to the cell surface at normal levels. Similarly, GluN1/GluN2B receptors in which the GluN2B subunit lacks most of its CTD are expressed at the cell surface^34^, whereas GluN1/GluN2C receptors lacking the CTD are expressed at the surface at reduced numbers^57^. Nevertheless, we cannot exclude the possibility that the CTD in the GluN3A subunit regulates other steps in the trafficking of GluN3A-containing NMDARs, including surface mobility and/or stability; here, however, we focused on the surface delivery of GluN1/GluN3A receptors containing mutations in the subunits’ glycine-binding sites.

In GluN1/GluN3A receptors, the glycine-binding site in GluN3A subunit activates the receptor, whereas the glycine-binding site in GluN1 subunit mediates the rapid desensitisation kinetics of glycine-induced currents^16–19^. Here, we analysed glycine-induced currents in order to measure the functional properties of various GluN1/GluN3A receptors containing mutations in the glycine-binding sites of both GluN1 and GluN3A subunits. We found that the changes in τ_w_ values for desensitisation of mutant GluN1/GluN3A receptors is consistent with previously measured changes of EC_50_ values for glycine binding to mutant GluN1/GluN2 receptors^17,54^. This finding is also consistent with previous studies that used mutant GluN1 subunits (e.g. GluN1-F484A subunit) to reduce the desensitisation kinetics of GluN1/GluN3A receptors^17,52,53,56,58,59^. The observed order of changes in EC_50_ values for the mutant GluN1-4a-F484A/GluN3A receptors based on peak current amplitude is similar to previous reports of steady-state currents^16,17^. Moreover, an analysis of peak currents enabled us to estimate the EC_50_ value for wild-type GluN1-4a/GluN3A receptors as well, which was previously not possible due to the virtually negligible steady-state currents when these receptors were expressed in *Xenopus* oocytes^16,17,60^. However, we observed an apparent rightward shift in the concentration-response curve for GluN1-4a/GluN3A receptors compared to previously reported estimates of glycine EC_50_ values for steady-state currents based on bell-shaped curves^17^ as well as to previously reported Kd values from binding studies using soluble LBDs^61^; this shift could be due to a variety of factors, including: *i*) decreased glycine affinity of the peak current compared to the steady-state current, *ii*) the fast desensitisation kinetics of GluN1/GluN3A receptors, and/or *iii*) the use of different expression systems. Furthermore, we found that both the GluN1/GluN3A-T825L and GluN1/GluN3A-Y605A receptors have similar desensitisation kinetics but markedly decreased glycine affinity compared to wild-type receptors. These findings support the conclusion that the glycine-binding site in GluN3A subunit is required for activating the GluN1/GluN3A receptors but does not contribute to the desensitisation of GluN1/GluN3A receptors. This conclusion is in agreement with recent elegant study using CGP-78608, that in addition revealed the presence of functional GluN1/GluN3A receptors in juvenile rat hippocampus^56^.

Our microscopy and quantitative assay data showed that analogous mutations in the glycine-binding sites of GluN1 and GluN3A subunits reduce the surface delivery of their respective NMDARs when expressed in both mammalian cell lines and hippocampal neurones. Given that endogenous GluN1, GluN2A-B, and GluN3 subunits are likely expressed in hippocampal neurones^12,21,22,56,62^, our data support the view that the structural changes in the glycine-binding sites in exogenous GluN1 and GluN3A subunits dominate the trafficking of NMDARs that include endogenous GluN subunits (for example, in the case of tri-heteromeric GluN1/GluN2/GluN3A receptors). Although the underlying mechanism by which mutations in the glycine-binding sites alters surface delivery is currently unknown, it is likely that the quality control machinery in the ER plays a role, as was shown previously for GluN1/GluN2 receptors as well as α-amino-3-hydroxy-5-methyl-4-isoxazolepropionic acid receptors (AMPARs)^48,49,63^. Glutamate may be present in the ER in the millimolar range^64,65^ and may be important for detecting properly assembled functional GluN1/GluN2 receptors and AMPARs via an ER-specific quality control mechanism. The precise concentration of glycine in the ER is unknown; however, given the role that glycine plays in various metabolic pathways^66^, it is likely present in sufficient amounts to interact with newly assembled receptors. In theory, a neurone could regulate the number of NMDARs at the cell surface by controlling the production of agonists such as glycine and D-serine or competitive antagonists such as kynurenic acid, which are found naturally in the mammalian CNS^2,67^. In addition, certain pharmacological compounds that act on the glycine-binding sites of GluN1 and/or GluN3A subunits may alter the surface expression of NMDARs, thereby providing a possible therapeutic strategy for certain pathological conditions. In this respect, it is important to note that treating cells with up to 1 mM glycine for 48 hours did not affect the surface expression of either wild-type or mutant GluN1/GluN3A receptors (Supplementary Fig. S4). This suggests that: *i*) intracellular glycine is already present at saturating concentrations in cultured cells, *ii*) high levels of extracellular glycine do not sufficiently alter the concentration of glycine within the ER, and/or *iii*) high levels of glycine do not regulate the intracellular processing of GluN1/GluN3A receptors.

Importantly, we also examined two putative pathogenic mutations in the LBD of the human GluN3A subunit. The N549S mutation is associated with the risk of nicotine dependence (rs75981117)^68^, and the D845N mutation has been classified by the UCSC browser as “clinically associated” (rs146060776). We found that the N549S mutation had no effect on the surface delivery or desensitisation kinetics of NMDARs and only slightly reduced the receptor’s affinity for glycine, consistent with the idea that the N549 residue in GluN3A subunit likely does not contribute directly to the subunit’s glycine-binding site. These conclusions are also in the agreement with our recent study which showed that the GluN3A-N549Q mutation (which should disrupt the predicted glycosylation of this residue) does not alter the surface delivery or desensitisation kinetics of the GluN1/GluN3A receptors^51^. In contrast, the D845N mutation drastically reduced the surface delivery of NMDARs, consistent with the high degree of homology between the rat and human GluN3A subunits (~89%) and the fact that we observed a similar reduction in the surface expression of the NMDARs containing the rat GluN3A-D845A subunit. Indeed, a growing number of reports have identified pathogenic mutations within GluN subunits, and new pathogenic mutations within the LBD ‒ particularly the glycine-binding site ‒ of both GluN1 and GluN3A subunits will likely be identified in future studies.

Taken together, our findings regarding the role of glycine-binding sites in GluN1 and GluN3A subunits in the surface delivery of NMDARs provide new previously overlooked insight into the physiological and pathophysiological regulation of these receptors in the mammalian CNS.

## Methods

### Molecular biology

Generation of the untagged rat GluN1-4a (GenBank: U08267.1; pcDNA1 vector), YFP-tagged rat GluN1-1a (GenBank: U08261; pcDNA3.1 vector)^69,70^, and GFP-tagged rat GluN3A (Gene ID: 191573; pCINeo vector)^30^ subunits have been described previously. The cDNA encoding the human GluN3A subunit was purchased from Origene, and the GFP-tagged human GluN3A (hGluN3A) construct was generated by cloning the human version of the *GRIN3A* gene into the rat GFP-GluN3A expression vector. The human version of the GluN1-4a subunit (hGluN1-4a) was generated by changing the four amino acid residues (N159S, R212K, I267L, M415L) that differ between the rat and human GluN1-4a subunits (differences in the signal peptides were ignored). All point mutations were introduced using the Quick-Change site-directed mutagenesis kit (Agilent Technologies) and were verified by DNA sequencing. K44A HA-dynamin 2 pcDNA3.1 (dynamin-K44A) was a gift from Sandra Schmid (Addgene plasmid # 34685; http://n2t.net/addgene:34685; RRID:Addgene_34685).

### Mammalian cell culture

COS-7 and HEK293 cells were obtained from ATCC and cultured in Opti-MEM I (Thermo Fisher Scientific) containing 5% fetal bovine serum (Thermo Fisher Scientific)^69,70^. We employed the COS-7 cells for microscopy as these cells were also employed previously in two relevant papers concerning this study^48,49^. In addition, COS-7 cells are larger than HEK293 cells which is advantagenous for performing localisation studies. For electrophysiology, we used HEK293 cells because they are (i.) smaller than the COS-7 cells that enables us to better compensate patch-clamp recordings, (ii.) routinely used for electrophysiology of NMDARs in most laboratories. Finally, we employed the HEK293 cells for quantitative assays to compare the surface delivery of GluN1/GluN3A receptors in both cell lines. HEK293 cells and COS-7 cells were transfected with 2 µl Lipofectamine 2000 (Thermo Fisher Scientific) plus 900 ng of total cDNAs encoding the GluN1 and GluN3A subunits. For electrophysiology, transfected cells were trypsinised and grown at low density; cells intended for microscopy and quantitative assays were grown without the trypsinisation step. The experiments were performed 24–72 h after transfection.

### Primary hippocampal neurones

All animal experiments were approved by the Animal Care and Use Committee of the Institute of Physiology of the Czech Academy of Sciences (CAS) and were conducted in accordance with the guidelines of the European Union directive 2010/63/EU. The Institute of Physiology CAS possesses the National Institutes of Health Statement of Compliance with Standards for Humane Care and Use of Laboratory Animals. Primary cultures of hippocampal neurones were prepared from embryonic day 18 Wistar rats and cultured in Neurobasal media supplemented with B-27 (Thermo Fisher Scientific) and L-glutamine (Thermo Fisher Scientific) using established protocols^69^. At DIV10, the neurones were transfected using Lipofectamine 2000 as described previously^69^.

### Microscopy

COS-7 cells grown in 12-well plates were transfected with a mixture of Lipofectamine 2000 and 2:1 ratios of cDNAs encoding the GluN1-4a and GFP-GluN3A subunits were used (to minimize occurrence of GFP-positive cells lacking the expression of GluN1-4a subunits). The YFP-GluN1 or GFP-GluN3A subunits present at the cell surface of live cells were labelled using rabbit anti-GFP as the primary antibody (AB3080P, 1:1000; Merck) and an anti-rabbit antibody conjugated to Alexa Fluor 555 (A21429, 1:1000; Thermo Fisher Scientific) or Alexa Fluor 647 (A21244, 1:1000; Thermo Fisher Scientific) for COS-7 cells and hippocampal neurones, respectively; the antibodies were diluted in blocking solution containing 3% normal goat serum (Thermo Fisher Scientific)^69,70^. The stained cells were then fixed in 4% paraformaldehyde (PFA; Sigma-Aldrich) in phosphate-buffered saline (PBS) for 20 min, and mounted using ProLong Gold Antifade reagent (Thermo Fisher Scientific). For labelling the total pool of GluN subunits, fixed cells were permeabilised with 0.25% Triton X-100 (Serva) in PBS for 5 min, and then incubated with anti-GFP followed by an anti-rabbit antibody conjugated to Alexa Fluor 488 (A11034, 1:1000; Thermo Fisher Scientific)^69,70^. Colocalisation experiments were performed on fixed COS-7 cells labelled with rabbit anti-GM130 antibody (G7295, 1:1000; Sigma-Aldrich) followed by an anti-rabbit antibody conjugated to Alexa Fluor 647 (1:1000). COS-7 cells and hippocampal neurones were imaged at room temperature using an Olympus Cell R microscope with a 60x/1.35 oil immersion objective (surface expression experiments on COS-7 cells) or a Leica SP8 confocal scanning microscope with a 63x/1.40 oil immersion apochromatic objective (colocalisation experiments on COS-7 cells; hippocampal neurons), respectively. The *z*-stack for all images was 0.3 µm, and the resolution was 1344 × 1024 pixels and 1024 × 1024 pixels for COS-7 cells and hippocampal neurones, respectively. The images were analysed using ImageJ 1.52N software (NIH) as described previously^69,71^. In brief, the surface and total fluorescence intensities of COS-7 cells were analysed on whole-cell areas. For hippocampal neurones, the fluorescence intensity of the total and surface signals was analysed on 5 separate 10-µm segments of secondary or tertiary dendrites per neurone. Prior to the intensity analysis, a *z*-stack projection was made with maximal intensity from the bottom of the cell to the top of the cell. Colocalisation of GluN subunit combinations with GM130 marker was analysed from single z-stack using an automated ImageJ macro to batch process the data. First, the background was subtracted using ImageJ function Subtract background with rolling factor set to 64. Mask of the entire cell area was generated from native GFP signal by thresholding. For GA area localisation a mask was generated from GM130 signal by thresholding. The thresholds were determined from fluorescent intensity histograms using ImageJ function Threshold with Otsu algorithm and the obtained thresholds were used for batch processing across the all data. A GM130-negative cell area was generated by subtraction of the GM130 mask from the GFP signal mask. The GA colocalisation was then calculated as a ratio of the average intensity of GFP signal through the generated GM130 mask over the average intensity of GFP signal through the GM130-negative cell area.

### Electrophysiology

Whole-cell patch-clamp recordings were performed on transfected HEK293 cells expressing GluN1/GluN3A receptors using an Axopatch 200B amplifier (Molecular Devices) as described previously^51,58^. The extracellular solution contained (in mM): 160 NaCl, 2.5 KCl, 10 HEPES, 10 glucose, 0.2 EDTA, and 0.7 CaCl_2_ (pH adjusted to 7.3 with NaOH). The intracellular solution contained (in mM): 125 gluconic acid, 15 CsCl, 5 BAPTA, 10 HEPES, 3 MgCl_2_, 0.5 CaCl_2_, and 2 ATP-Mg salt (pH adjusted to 7.2 with CsOH). CGP-78608 (Tocris) was prepared according to manufacturer’s instructions. Glass patch pipettes (3–6 MΩ tip resistance) were prepared using a model P-97 micropipette puller (Sutter Instrument Co.). A microprocessor-controlled multi-barrel rapid perfusion system (with a time constant for solution exchange around the cell of ~10 ms) was used to apply the extracellular solutions^57^. All electrophysiology experiments were performed at room temperature. pCLAMP 9 software (Molecular Devices) was used to record and analyse the glycine-induced currents at a membrane potential of −60 mV.

### Calculation of glycine potency

Data were analysed using SigmaPlot 10.0 (Systat Software, Inc.). The normalised peak concentration-response data for each HEK293 cell were best-fitted using the following equation:

1 $$I={I}_{max}/(1+{({{\rm{EC}}}_{50}/[{\rm{Glycine}}])}^{h}),$$

where *I*_*max*_ is the maximum peak current in response to glycine, EC_50_ is the glycine concentration that elicited the half-maximal response, [Glycine] is glycine concentration, and *h* is the apparent Hill coefficient. The peak current (*I*) through GluN1/GluN3A receptors was elicited by applying glycine at concentration of 10–10,000 μM and was normalised to the maximum peak current recorded in that cell.

### Quantitative assay of surface expression

HEK293 cells grown in 12-well plates were transfected with a mixture of Lipofectamine 2000 and equal amounts of cDNA vectors containing dynamin-K44A and/or GluN subunits, as described^52^. After 38–40 hr, cells were fixed for 20 min in 4% PFA in PBS and incubated for 60 min in PBS containing 0.2% bovine serum albumin (BSA) without (surface expression) or with (total expression) 0.1% Triton X-100. Next, cells were incubated in primary rabbit anti-GFP antibody (AB3080P; Merck; 1:500 for surface expression and 1:1000 for total expression) and then with secondary horseradish peroxidase-conjugated donkey anti-rabbit IgG (NA934V; GE Healthcare; 1:1000), both diluted in PBS with 0.2% BSA for 1 hr. The color reaction was done with ortho-phenylenediamine (OPD; 0.4 mg/ml) dissolved in phosphate-citrate buffer containing sodium phosphate (both from Merck) for 30 min (surface expression) or 15 min (total expression) and was terminated with 3 M HCl. The optical density was determined at 492 nm using a Personal Densitometer SI (GE Healthcare). Data were obtained from three independent experiments (each included two different wells for surface or total expression for each GluN subunit combination). The background signal was subtracted and data were normalized to average data obtained from the cells expressing control GluN1-4a/GFP-GluN3A receptors.

### Statistical analysis

The quantitative microscopy/assays and electrophysiology data were compared to the respective wild-type group, and summary data are presented as the mean ± the standard error of the mean (SEM). Group differences were analysed using a Student’s t-test or a one-way ANOVA followed by the Dunnett’s Method using SigmaStat 3.5 (Systat Software, Inc.), and differences with a *p*-value < 0.05 were considered significant.

## Supplementary information

Supplementary figures

## Data Availability

All materials, data, and associated protocols will be promptly made available to readers upon request without undue qualifications for material transfer agreements.
