# Supplementary material for: Structural features in the glycine-binding sites of the GluN1 and GluN3A subunits regulate the surface delivery of NMDA receptors
Source: Sci Rep. 2019 Aug 23;9:12303. doi: 10.1038/s41598-019-48845-3 (PMC6707325; doi:10.1038/s41598-019-48845-3)
Supplement: Supplementary file 1 — Supplementary figures [file 41598_2019_48845_MOESM1_ESM.pdf]

Supplementary data

Structural features in the glycine-binding sites of the GluN1 and GluN3A subunits regulate the surface delivery of NMDA receptors

Kristyna Skrenkova [1,2], Katarina Hemelikova [1,2], Marharyta Kolcheva [1,2,3], Stepan Kortus [1], Martina Kaniakova [1,2], Barbora Krausova [1,2], Martin Horak [1,2*]

[1] Institute of Experimental Medicine of the Czech Academy of Sciences, Videnska 1083, 14220 Prague 4, Czech Republic

[2] Institute of Physiology of the Czech Academy of Sciences, Videnska 1083, 14220 Prague 4, Czech Republic

[3] Department of Physiology, Faculty of Science, Charles University in Prague, Albertov 6, 12843 Prague 2, Czech Republic

Supplementary Figure S1

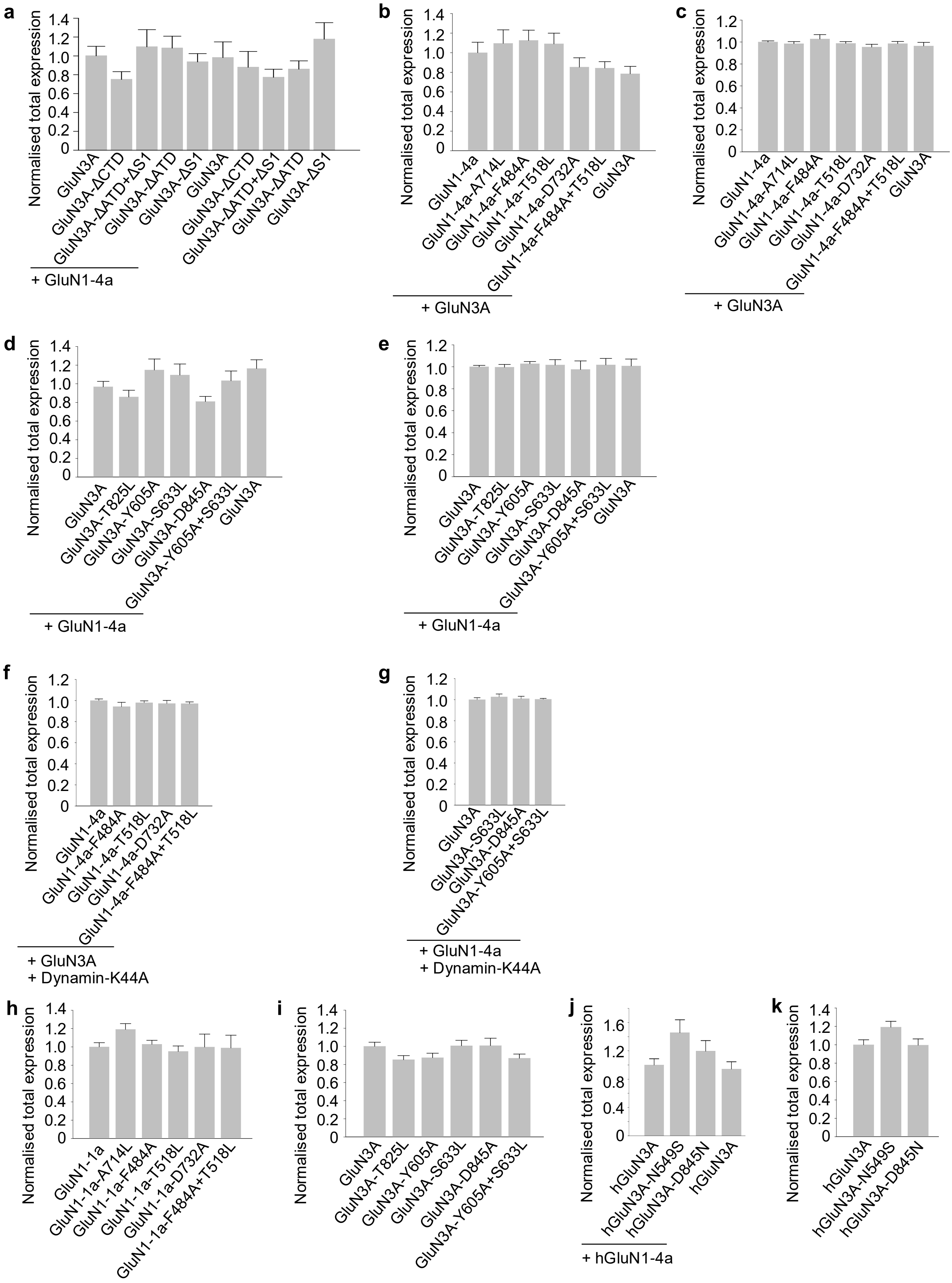

Figure S1. The total expression levels were not significantly different among the studied GluN subunit combinations. **(a)** corresponds to Fig. 1c; **(b)** corresponds to Fig. 2c; **(c)** corresponds to Fig. 2d; **(d)** corresponds to Fig. 3c; **(e)** corresponds to Fig. 3d; **(f)** corresponds to Fig. 4a; **(g)** corresponds to Fig. 4b; **(h)** corresponds to Fig. 5b; **(i)** corresponds to Fig. 5d; **(j)** corresponds to Fig. 6d; **(k)** corresponds to Fig. 6j. (p>0.05; ANOVA).

Supplementary Figure S2

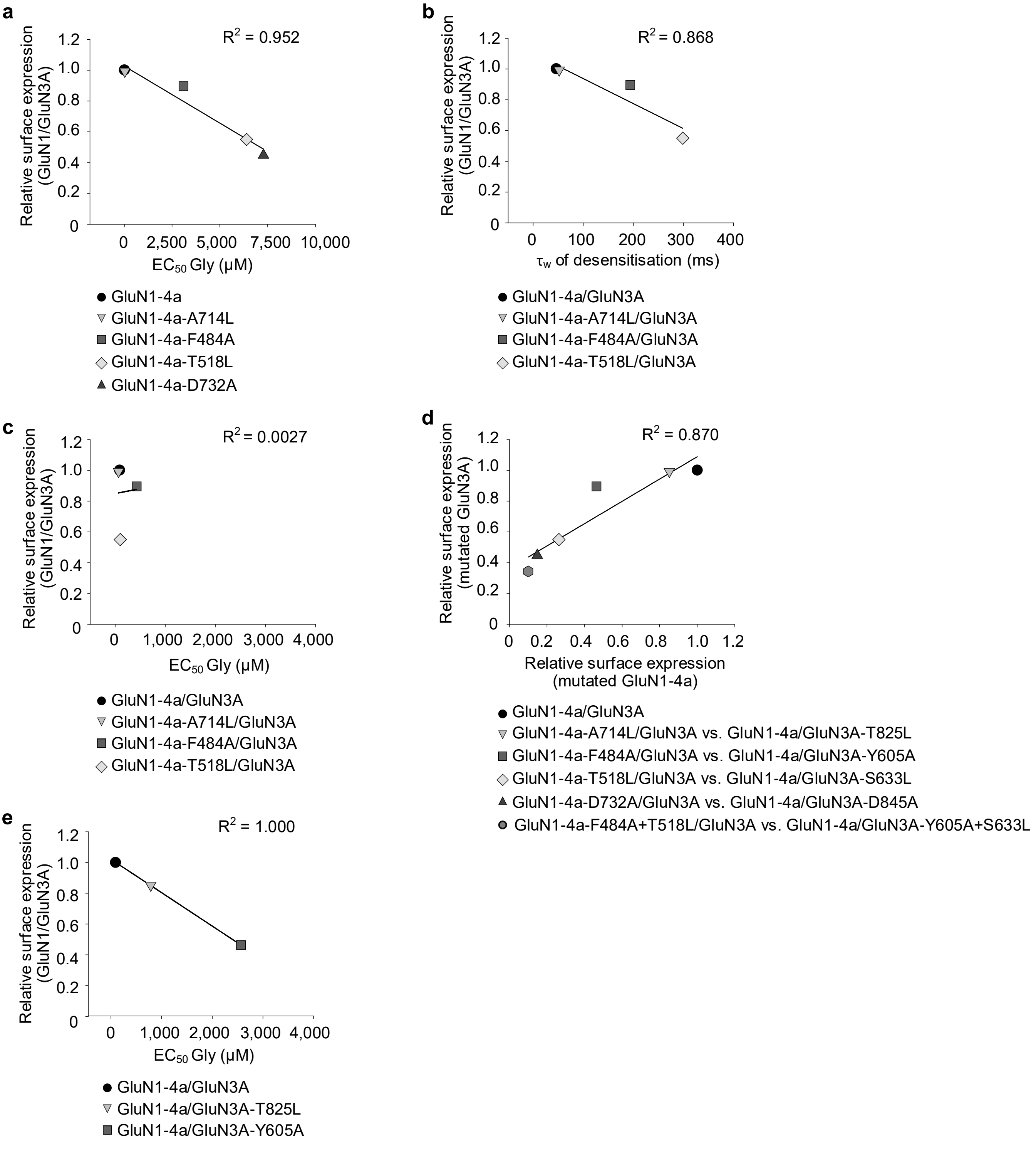

Figure S2. The correlation analysis of surface expression of the mutated GluN1/GluN3 receptors. (a) Correlation of relative surface expression shown in Fig. 2d with the EC_{50} values for glycine reported in the following studies: GluN1 (reported EC_{50} value at the GluN1/GluN2A receptors was 1.2 µM); GluN1-A714L mutation (reported EC_{50} value at the GluN1/GluN2A receptors was 27 µM); the GluN1-F484A mutation (reported EC_{50} value at the GluN1/GluN2A receptors was 3100 µM); the GluN1-T518L mutation (reported EC_{50} value at the GluN1/GluN2A receptors was 6400 µM) [1]; the GluN1-D732A mutation (reported EC_{50} value at the GluN1/GluN2B receptors was 7284 µM) [2]. **(b)** Correlation of relative surface expression from Fig. 2d with the τ_w of desensitisation (1000 µM glycine) shown in Fig. 2f. **(c)** Correlation of relative surface expression shown in Fig. 2d with the EC_{50} values shown in **Table 1**. **(d)** Correlation of relative surface expression shown in Fig. 2d with the relative surface expression shown in Fig. 3d. **(e)** Correlation of relative surface expression shown in Fig. 3d with the EC_{50} values shown in **Table 1**. The calculated coefficient of determination (R^2) is shown in each graph.

Supplementary Figure S3

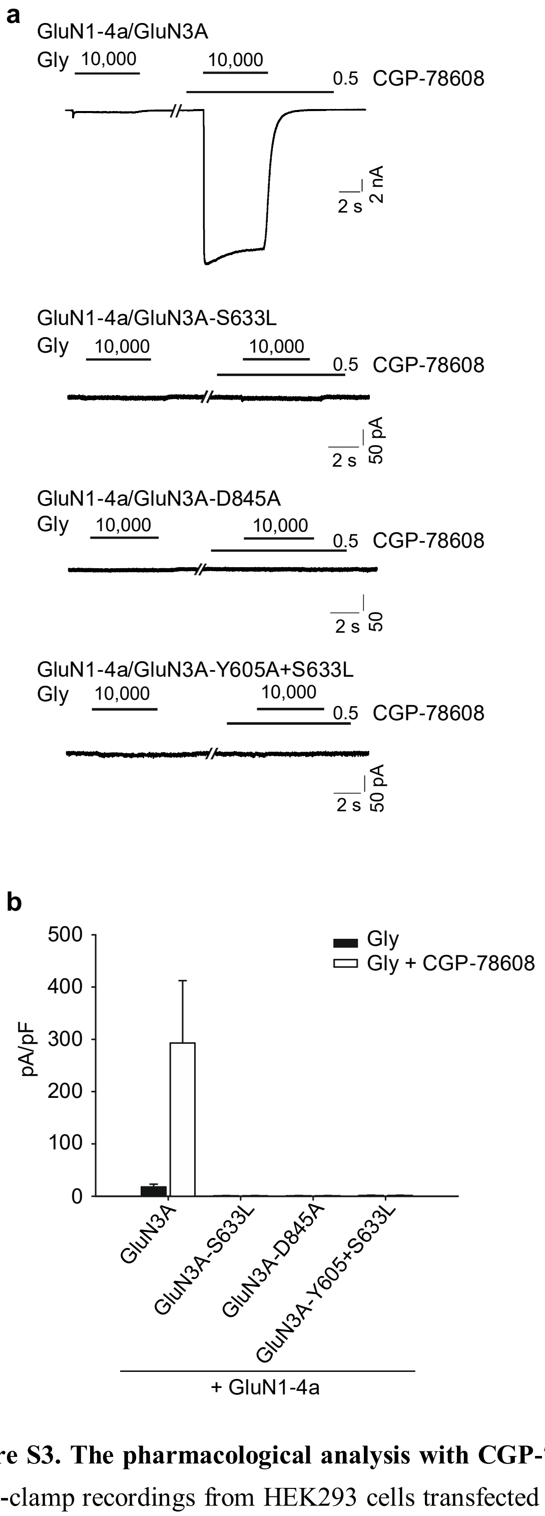

Figure S3. The pharmacological analysis with CGP-78608 at the GluN1/GluN3A receptors. (a) Representative whole-cell patch-clamp recordings from HEK293 cells transfected with the indicated wild-type or mutant GluN1-4a/GluN3A receptors at a membrane potential of -60 mV. Currents were elicited by applying 10,000 μM glycine; 0.5 μM CGP-78608 was applied as indicated. **(b)** Summary of current densities (pA/pF) obtained from the HEK293 cells expressing the indicated GluN1-4a/GluN3A receptors (n≥6 cells per group).

Supplementary Figure S4

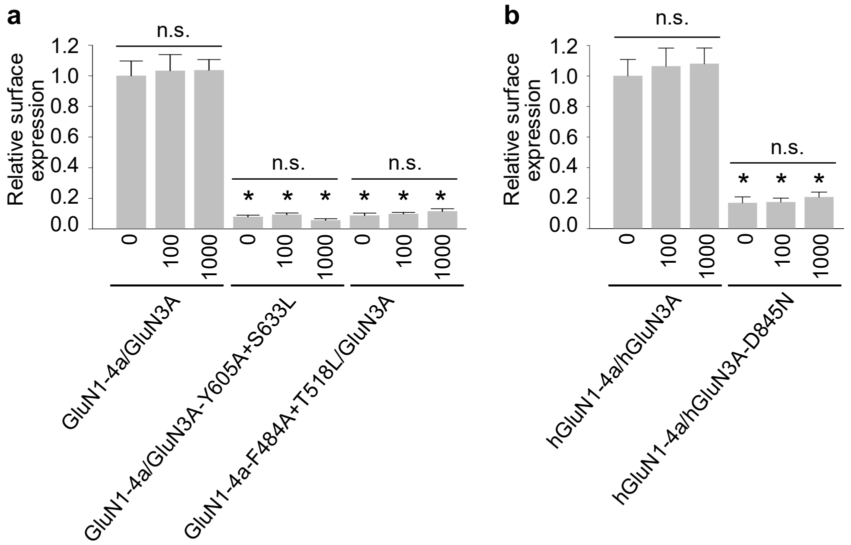

Figure S4. Chronic application of glycine does not affect the surface expression of wild-type or mutant GluN1/GluN3A receptors. COS-7 cells were transfected with the indicated rat (a) or human (b) GluN1 and GluN3A subunits. The cells were then incubated for 48 h in the absence or presence 100 μM or 1000 μM glycine, after which surface and total subunits were measured using fluorescence microscopy (n≥32 cells per group); *p<0.05 vs. the respective GluN1-4a/GluN3A (a) or hGluN1-4a/hGluN3A (b) receptor group (ANOVA).

Supplementary references

1 Kvist, T., Greenwood, J. R., Hansen, K. B., Traynelis, S. F. & Brauner-Osborne, H. Structure-based discovery of antagonists for GluN3-containing N-methyl-D-aspartate receptors. *Neuropharmacology* **75**, 324-336, doi:S0028-3908(13)00360-2 [pii]10.1016/j.neuropharm.2013.08.003 (2013).

2 Williams, K., Chao, J., Kashiwagi, K., Masuko, T. & Igarashi, K. Activation of N-methyl-D-aspartate receptors by glycine: role of an aspartate residue in the M3-M4 loop of the NR1 subunit. *Mol Pharmacol* **50**, 701-708 (1996).
